# Delivering the Gut to the Brain: Drug Delivery Strategies for Microbiota-Derived Therapeutics in Depression

**DOI:** 10.3390/pharmaceutics18091112

**Published:** 2026-09-03

**Authors:** Yohan Seo, Chul Soon Park

**Affiliations:** 1 Department of Physiology, College of Medicine, Dongguk University, Gyeongju 38066, Republic of Korea; 2 Department of Bio-Nanomaterials, Bio Campus of Korea Polytechnics, Nonsan 32943, Republic of Korea

**Keywords:** depression, microbiota–gut–brain axis, drug delivery, colon targeting, bacterial extracellular vesicles, blood–brain barrier

## Abstract

Major depressive disorder remains a leading cause of disability worldwide, and the limited efficacy and delayed onset of conventional antidepressants have intensified interest in the microbiota–gut–brain axis as a source of therapeutic targets. Microbiota-associated candidates—short-chain fatty acids, bile acid and tryptophan metabolites, and neuroactive amines—show mood-relevant activity, yet almost none has reached the clinic. This review reframes that gap as a delivery problem. Rather than treating entry into the central nervous system as a universal requirement, we distinguish strategies intended for local intestinal, peripheral systemic, and direct central action, and we argue that delivery is a major but not exclusive translational bottleneck. We outline the barriers these agents face—upper gastrointestinal loss, poor colonic targeting, rapid metabolite turnover, first-pass exposure, and the blood–brain barrier—and synthesize delivery strategies across two fronts. Colon-targeted systems are technically established but have been validated for non-depression indications, whereas brain-directed approaches—bacterial extracellular vesicles, detoxified membrane-coated carriers, receptor-mediated transcytosis, and intranasal routes—reach the brain mainly in selected preclinical models. We foreground a paradox: microbial extracellular vesicles are at once one of the better-documented bio-derived routes for brain exposure in preclinical studies and prominent drivers of neuroinflammation, which defines a risk–opportunity continuum. We close with a route-specific validation roadmap encompassing quantitative exposure, target engagement, chronic efficacy, and safety.

## 1. Introduction

Major depressive disorder (MDD) is among the leading causes of disability worldwide [1], and the shortcomings of current pharmacotherapy have become increasingly difficult to ignore. First-line agents such as selective serotonin and serotonin–norepinephrine reuptake inhibitors achieve full remission in only about one-third of patients, act with a delayed onset of two to four weeks, and frequently produce adverse effects that undermine adherence [2]. These limitations have driven a search for mechanistically distinct interventions, and over the past decade the microbiota–gut–brain axis has emerged as one of the most actively investigated sources of new therapeutic targets [3,4,5]. Foundational germ-free and gnotobiotic studies established that the microbiota shapes stress reactivity, anxiety-like behavior, and memory [6,7,8].

Much of that investigation has centered on which molecules matter and why. A now-substantial literature implicates microbiota-derived molecules—short-chain fatty acids (SCFAs), bile acid derivatives, tryptophan metabolites, and neuroactive amines such as GABA and serotonin—as mediators of mood-relevant signaling along the gut–brain axis [9,10,11]. Comprehensive reviews have mapped the associations between dysbiosis, intestinal barrier dysfunction, and depression [12,13,14,15,16] and cataloged microbiota-targeted strategies including dietary intervention, fecal microbiota transplantation, and probiotic, prebiotic, synbiotic, and postbiotic supplementation [11,17,18,19,20,21,22].

A different and comparatively underexamined question stands between these candidates and the clinic: not what to deliver, but how to deliver it. A molecule with genuine antidepressant potential is of little use if it is degraded before reaching the colonic microbial community that generates or responds to it, if it is cleared from the circulation within minutes, or if it cannot traverse the blood–brain barrier (BBB) to reach central targets. Many—though not all—of the leading microbiota-derived candidates are pharmaceutically fragile in precisely these ways, and the route each must travel depends on whether its mechanism is local intestinal, peripheral systemic, or directly central. This review therefore reframes the field through the lens of drug delivery, treating delivery not as an implementation detail but as a major, underexamined translational bottleneck—one of several rate-limiting challenges, and the one this review takes as its focus.

This reframing is deliberately distinct from adjacent bodies of work. Mechanistic reviews of the gut–brain axis in depression concentrate on pathophysiology and on live-biotherapeutic or dietary interventions, largely setting aside formulation science. A separate and excellent literature has examined the pharmaceutical delivery of microbial metabolites—enteric encapsulation, esterification to dietary fiber, prodrugs, and nanoformulation—but does so in a disease-agnostic manner weighted toward gastrointestinal, metabolic, and immune indications, and stops at the gut or the systemic circulation rather than the brain [23]. A third strand reviews extracellular-vesicle and nose-to-brain delivery for central nervous system (CNS) disorders in general (e.g., [24]), without a microbiota focus or a depression focus. Recent reviews have addressed microbiota-derived EVs in psychiatric and neurological disorders, the formulation of microbial metabolites, and intranasal EV delivery separately, but these studies remain largely disconnected. The distinctive contribution of the present review is to integrate them according to intended site of action, delivery route, and the level of depression-specific evidence, and to confront a paradox that cuts across them: microbial extracellular vesicles (EVs), which have demonstrated brain exposure in selected preclinical models, have been characterized overwhelmingly as agents of neuroinflammation rather than as therapeutic carriers.

We proceed as follows. We first summarize, briefly and by reference, the microbiota-derived molecules implicated in depression (Section 2), then define the pharmaceutical barriers that confront them—gastric and enzymatic degradation, poor colonic targeting, the short half-life of the free metabolites, and the BBB (Section 3). Section 4, the core of this review, synthesizes delivery strategies across two fronts: colon-targeted systems that are already maturing in gastrointestinal disease, and emerging routes to the brain. We then examine the central challenge of the field—repurposing pathological microbial–EV routes into safe carriers—and the caveats that honesty demands (Section 5), before mapping future directions toward clinical translation (Section 6).

### Scope, Terminology, and Literature Identification

In this review, “microbiota-derived therapeutics” refers to therapeutic molecules or biological materials produced directly by gut microorganisms or through host–microbial co-metabolism. Three categories are distinguished and kept distinct throughout. Category 1, microbiota-derived therapeutics, comprises short-chain fatty acids, microbial indoles, and other microbial or host–microbial co-metabolites, together with bacterial extracellular vesicles. Category 2, microbial extracellular vesicles, denotes bacteria-derived vesicles including outer membrane vesicles; these are treated as a distinct microbial carrier class within Category 1, and their pathogen-associated molecular pattern content and immunogenicity are central to their evaluation. Category 3, non-microbial bio-derived vesicles, comprises mammalian-, milk-, plant-, and microalgal-derived extracellular vesicles. These are not considered microbiota-derived therapeutics and are included only as non-microbial comparator platforms where they provide transferable formulation or delivery principles; they are labeled as such wherever they appear, including in the comparative brain-delivery evidence table. Evidence is interpreted throughout according to its proximity to the intended clinical application: human depression evidence is considered the most directly relevant, followed by animal depression models, non-depression CNS studies, and finally non-CNS or engineering proof-of-concept studies. This hierarchy indicates inferential distance rather than a formal GRADE-type assessment. This article is a critical narrative review rather than a systematic review. PubMed, Scopus, and Web of Science were searched from database inception to 31 May 2026 (final search date), restricted to English-language, peer-reviewed publications; no lower date limit was applied. The principal Boolean strings were: (depression OR “major depressive disorder” OR “depressive-like behavio*”) AND (microbiot* OR microbiome OR “short-chain fatty acid*” OR butyrate OR “bile acid*” OR indole OR kynurenine OR GABA OR serotonin); (“colon-target*” OR “colonic delivery” OR “enteric coating” OR “pH-responsive” OR “azo-polymer” OR prodrug) AND (formulation OR nanoparticle OR “drug delivery”); (“bacterial extracellular vesicle*” OR “outer membrane vesicle*” OR OMV OR “bacterial membrane-coated”) AND (“blood–brain barrier” OR brain OR “central nervous system”); and (intranasal OR “nose-to-brain” OR “focused ultrasound”) AND (“extracellular vesicle*” OR exosome). Reference lists of retrieved reviews were hand-searched for additional primary studies. Records were prioritized when they reported (i) a defined delivery vehicle or formulation, (ii) an administration route, and (iii) an in vivo outcome; conference abstracts, non-peer-reviewed preprints, and studies without an identifiable delivery system were excluded. Where several reports supported the same point, primary experimental studies were cited in preference to reviews. Evidence is characterized throughout as in vitro, animal (non-depression), animal (depression), human (non-depression), or human depression, and the brain-delivery evidence table records this grading for brain-delivery claims.

## 2. Microbiota-Derived Molecules Implicated in Depression

Because this review concerns delivery rather than mechanism, we treat the candidate molecules only briefly—as the payloads whose transport the later sections must solve. Comprehensive accounts of their biology and their associations with depression are available elsewhere [11,20].

Four classes recur. Short-chain fatty acids (SCFAs)—principally acetate, propionate, and butyrate, produced by bacterial fermentation of dietary fiber—are the most studied [25,26]; they are depleted in some patients with MDD [27] and exert antidepressant-relevant effects through histone deacetylase inhibition [28,29], free fatty acid receptor signaling [30,31], and modulation of microglial [32] and blood–brain barrier function [33,34], with butyrate the most frequently implicated [11,25]. Bile acid derivatives, generated when gut bacteria chemically transform host primary bile acids, form a second class whose altered profiles have been linked to depression and that can modulate blood–brain barrier permeability [11,35]. Tryptophan metabolites—indole derivatives and kynurenine-pathway products of microbial and host co-metabolism—connect the microbiome to serotonergic and neuroinflammatory pathways relevant to mood, including aryl-hydrocarbon-receptor signaling in astrocytes and microglia [36,37,38,39]. Finally, bacteria produce or induce neuroactive amines, including γ-aminobutyric acid (GABA) [40,41] and serotonin (5-HT) [42,43,44], that participate in gut–brain signaling [20,45,46,47].

Two features of this inventory matter for what follows. First, these are chemically heterogeneous agents—small acids, steroid derivatives, amino-acid metabolites, and biogenic amines—so no single delivery solution fits all, which is why the strategies of Section 4 are correspondingly varied. Second, and more consequentially, most act at sites that are pharmacologically hard to reach: the distal gut, where they are generated and where much of their receptor biology resides, or the brain, beyond the blood–brain barrier. It is this mismatch—between where these molecules are promising and where they can currently be delivered—that Section 3 and Section 4 address. Table 1 summarizes these payload classes, intended sites of action, principal pharmacokinetic barriers, and candidate formulation strategies.

**Table 1:** Therapeutic payloads, their intended site of action, and delivery requirements. Whether a candidate must reach the brain depends on its mechanism; many act locally or peripherally. BBB, blood–brain barrier; CNS, central nervous system; EV, extracellular vesicle; OMV, outer membrane vesicle; PK, pharmacokinetic; RES, reticuloendothelial system; SCFA, short-chain fatty acid; 5-HT, serotonin; GABA, γ-aminobutyric acid.

Payload (Class)	Intended Site of Action	CNS Entry Required?	Principal PK Barrier	Candidate Formulation	Depression Evidence
Butyrate (SCFA)	Colon-local; peripheral; possibly central	Mechanism-dependent	Proximal absorption; rapid first-pass metabolism	Enteric coating; ester/prodrug (tributyrin); polymer nanocarrier	Mainly preclinical
Propionate/acetate (SCFA)	Colon-local; peripheral	Usually not established	Rapid turnover; proximal absorption	Inulin–propionate ester; sustained release	Preclinical/mechanistic
Secondary bile acids	Intestinal; systemic	Not established	Enterohepatic cycling	Targeted colonic release	Associative
Indole/kynurenine metabolites	Immune; possibly central (AhR)	Compound-specific	Rapid metabolism	Prodrug; nanoparticle	Preclinical
GABA	Vagal; peripheral	Direct CNS entry limited	Poor BBB penetration	Local or CNS-targeted derivative	Preclinical
Serotonin (5-HT)	Peripheral (enteric)	Peripheral 5-HT does not readily cross the BBB	Rapid metabolism	Indirect (precursor/modulatory) strategy	Mechanistic
Bacterial EV/OMV (cargo carrier)	Systemic → possibly central	Vehicle designed for CNS access	RES uptake (liver/spleen); immunogenicity	Detoxified/engineered EV; membrane-coated NP	Preclinical (rodent)

## 3. Barriers to Delivery

Before surveying what delivery technology can achieve, it is worth stating plainly what it must overcome. Crucially, delivery across the microbiota–gut–brain axis is not a single, universal sequence. The route a candidate must travel depends on where its therapeutic mechanism resides: a locally acting intestinal payload, a peripherally acting metabolite or immune modulator, and a directly central agent face different—and only partly overlapping—barriers. With that qualification, four obstacles recur, and any given candidate encounters the subset relevant to its intended site of action.

Gastric and enzymatic degradation. Most microbiota-derived candidates are intended to act in the distal gut or beyond, yet oral administration first exposes them to the stomach’s acidic environment and to the proteases, lipases, and esterases of the upper gastrointestinal tract. Short-chain fatty acids and their salts are absorbed proximally or neutralized before reaching the colonic microbial community, and peptide- or amine-based agents are especially vulnerable to enzymatic cleavage [48]. Any effective platform must therefore incorporate protection—an acid-resistant coat, a covalent linkage, or an encapsulating matrix—that survives the upper tract intact. Even nanoparticle carriers must then contend with the continuously secreted, rapidly shed intestinal mucus layer, which traps and clears particles before they reach the epithelium [49,50].

Poor colonic targeting. Even an agent that survives transit must be released in the right place. The therapeutic logic of many microbiota-derived molecules depends on delivery to the distal gut, where the relevant microbial density and much of the responsive receptor biology are concentrated; premature release in the small intestine squanders the dose and can provoke off-target effects. Achieving spatial specificity requires release triggers keyed to features that distinguish the colon—its higher luminal pH and its distinctive microbial enzyme activity—rather than simple time- or diffusion-based release.

Short half-life and instability of the free metabolites. A defining pharmaceutical weakness of this class is that the active molecules are often cleared or metabolized within minutes of liberation [51]. This is not a peripheral inconvenience but a central design constraint: it is the reason free-metabolite supplementation in preclinical work so often relies on high, frequently repeated doses that are difficult to translate clinically. The human inulin propionate ester study is illustrative—covalent tethering of propionate to a fermentable inulin backbone was required precisely because free propionate would not reach the colon in usable quantity, and even then delivery had to be engineered against rapid turnover [52]. Sustained or triggered release, rather than bolus delivery, is thus a prerequisite for a viable product.

The blood–brain barrier. For any candidate whose therapeutic effect depends on a central action, the final and most formidable barrier is the BBB, whose tight junctions and efflux transporters exclude essentially all large or charged molecules and the great majority of small ones [53,54]. This barrier is what separates the comparatively tractable problem of gut delivery from the still-open problem of brain delivery, and it is the reason Section 4 must treat the two destinations as fundamentally different engineering tasks. Notably, microbiota-derived metabolites themselves influence barrier integrity: alterations in the gut microbiota and in microbial short-chain fatty acid availability have been associated with impaired BBB integrity, whereas short-chain fatty acids such as propionate can reinforce endothelial barrier function [33,34]. Importantly, unlike glioma or acute neurovascular injury, depression does not present a reliably or uniformly disrupted BBB. Emerging human neuroimaging indicates regional barrier alterations in subsets of patients, particularly those with inflammatory phenotypes, but their magnitude and therapeutic relevance remain unresolved—so the barrier can be assumed neither uniformly intact nor dependably permeable, a point to which we return in Section 5.

These barriers are best understood not as a single fixed sequence every candidate must run, but as a menu from which each therapeutic draws according to its intended site of action; the delivery strategies that follow are answers to specific subsets of them.

Framed in biopharmaceutical terms, these obstacles reduce to a small number of measurable quantities that any candidate formulation must eventually report. Oral exposure is governed by the interplay of solubility and permeability, luminal and enzymatic stability, regional transit time, and the fraction escaping presystemic (gut-wall and hepatic) metabolism, which together determine bioavailability; for short-chain fatty acids the limiting term is not gastric degradation but proximal absorption followed by extensive first-pass extraction, whereas for peptidic or vesicular payloads enzymatic and colloidal stability dominate. Systemic exposure is then set by clearance, volume of distribution, plasma-protein and lipoprotein binding, and—for particulate carriers—opsonization and mononuclear-phagocyte-system uptake, which typically direct most an intravenous vesicle dose to liver and spleen. Central exposure must be expressed not as the presence of signal in brain but as an unbound brain-to-plasma partition coefficient or an equivalent quantitative measure obtained after vascular perfusion or capillary depletion. The formulation toolbox available to address these terms is correspondingly broad and now well established in pharmaceutics: enteric and time- or microbiota-triggered polymer coatings; matrix and reservoir systems giving zero-order or pulsatile release; covalent prodrugs and polymer–drug conjugates; lipid-based systems including liposomes, solid lipid nanoparticles, nanostructured lipid carriers, and lipid nanoparticles; biodegradable polymeric particles such as PLGA; mucoadhesive versus mucus-penetrating (PEGylated) surface designs; in situ gelling and thermosensitive vehicles for nasal administration; and spray-drying or lyophilization with cryoprotectants for solid-state stabilization. Selecting among them is a matter of matching the release trigger and the exposure requirement to the intended site of action, and of reporting the standard descriptors—particle size, polydispersity index, zeta potential, encapsulation efficiency and drug loading, in vitro release kinetics in biorelevant media, and storage stability—that make such comparisons possible. These descriptors are, at present, reported only sporadically in the microbiota-derived literature, and their absence is itself an obstacle to translation.

## 4. Delivery Strategies

**Figure 1:**
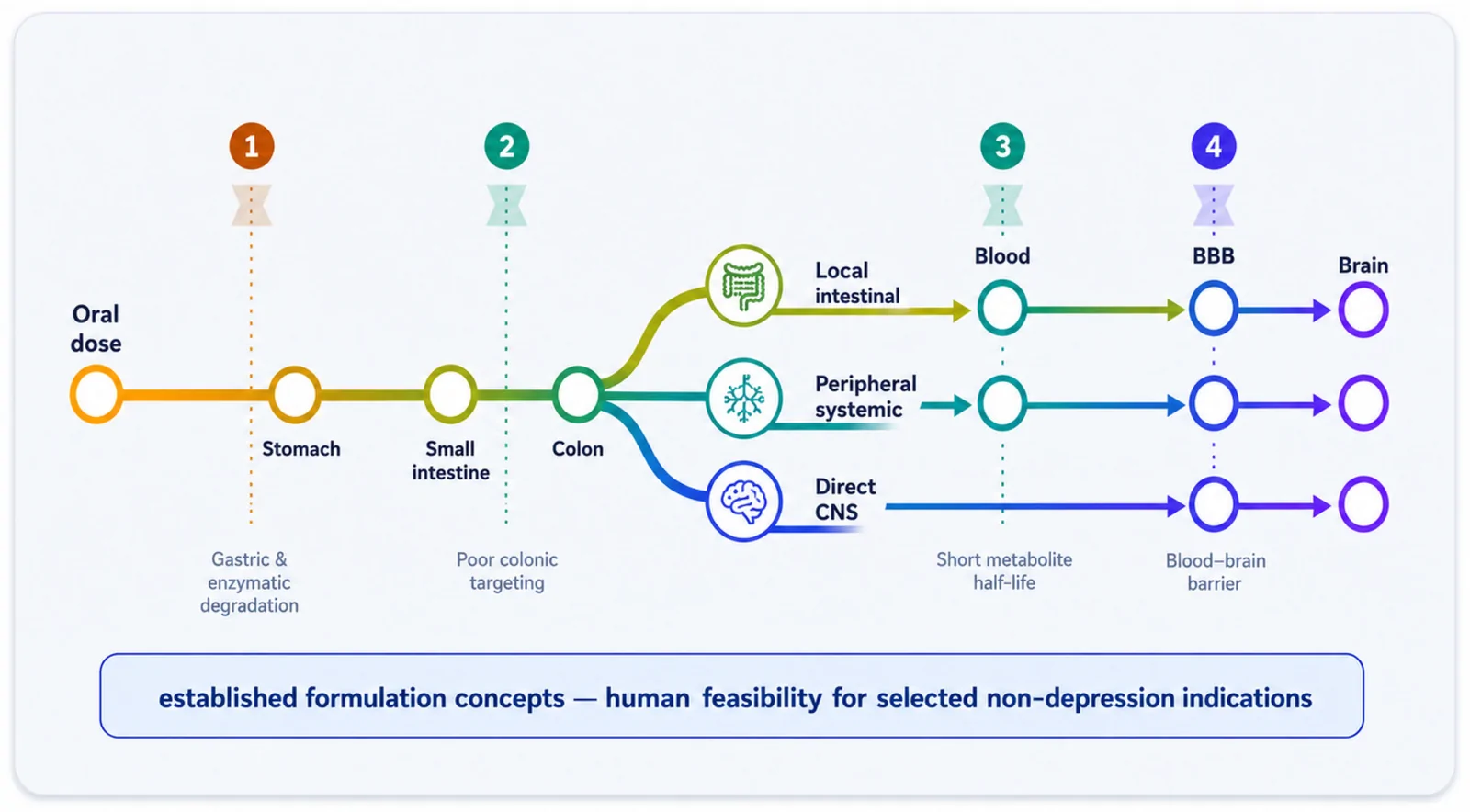
The two-front delivery problem. The route a microbiota-derived therapeutic must travel depends on where it acts. From the colon, three intended destinations diverge: local intestinal action (on epithelial, enteroendocrine, immune, and vagal targets), peripheral systemic action (via portal and systemic exposure), and direct central action requiring transport across or around the blood–brain barrier. Colon-targeted delivery addresses upper-gastrointestinal loss, colonic targeting, and metabolite turnover, and its formulation concepts have demonstrated human feasibility for selected non-depression indications; brain-targeted delivery is required only for the central route and remains an emerging front whose enabling demonstrations lie outside depression. BBB, blood–brain barrier; CNS, central nervous system. Colors distinguish schematic routes and stages only and do not encode quantitative values.

If Section 3 establishes that microbiota-derived antidepressant candidates are pharmaceutically fragile, this section asks what can be done about it. The available strategies fall naturally into two groups defined by their destination. Colon-targeted delivery—getting an intact agent to the distal gut, where the microbial community and much of the relevant receptor biology reside—is a comparatively mature engineering problem, with several platforms already validated in humans and in animal models of gastrointestinal and systemic disease. Brain-targeted delivery—getting an agent, or a signal derived from it, across the BBB to central targets—is far less developed and is where the decisive translational questions for depression lie. We treat the two in turn, and we are explicit throughout about a recurring gap: nearly all of the delivery systems below were developed and validated for indications other than depression, so their relevance here is as transferable engineering, not as established antidepressant delivery (Figure 1).

### 4.1. Colon-Targeted Delivery: A Maturing Toolbox

**Figure 2:**
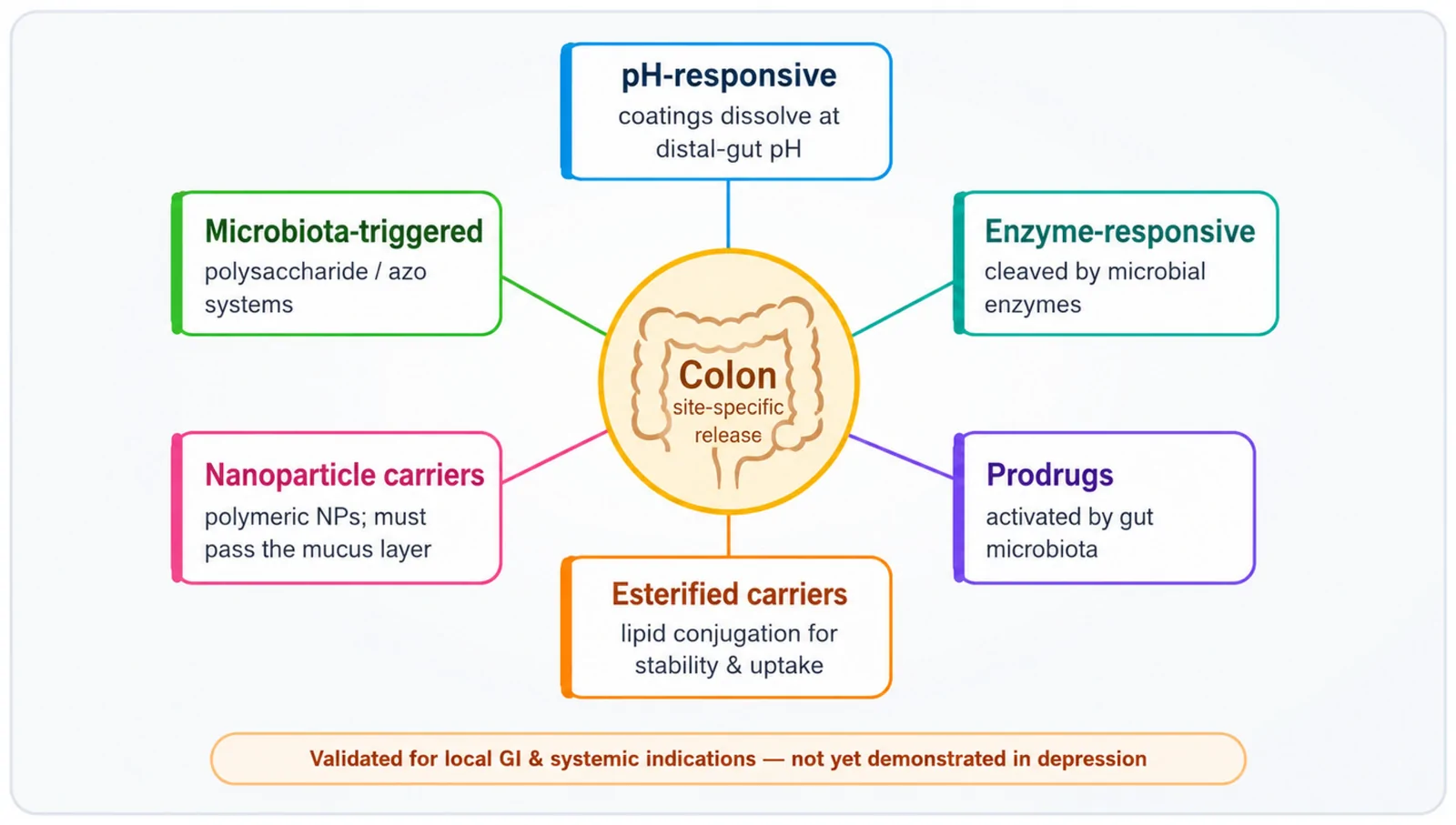
Colon-targeted delivery: a maturing toolbox. Strategies for achieving site-specific release in the distal gut, where the responsive microbial community is concentrated: pH-responsive and enzyme-responsive coatings, prodrug and carrier-integrated designs, esterification to fermentable dietary fiber, polymeric nanoparticle carriers (which must additionally negotiate the intestinal mucus barrier), and microbiota-triggered polysaccharide or azo-linked systems. These platforms are validated for local gastrointestinal and systemic indications but not yet for depression. GI, gastrointestinal.

The colon is an attractive delivery destination for microbiota-derived agents because it is where the producing and responding microbial community is concentrated and where locally acting metabolites such as butyrate exert their best-characterized effects [55]. The engineering challenge is to shepherd a labile small molecule past the acidic stomach and the enzyme-rich small intestine so that release occurs specifically in the distal gut. Several approaches have converged on this goal, including pH- and enzyme-responsive coatings, prodrugs, nanoparticle carriers, and microbiota-triggered polysaccharide or azo-linked systems that exploit the colon’s dense bacterial enzyme activity for site-specific release [56,57,58]. Polymeric nanoparticle carriers for oral delivery must additionally negotiate the mucus barrier, motivating mucus-penetrating and mucoadhesive particle designs [59,60]. These colon-targeting approaches are summarized in Figure 2.

pH- and enzyme-responsive coatings exploit the rise in luminal pH and the distinctive microbial enzyme activity of the colon as release triggers. A representative and instructive example is the surface engineering of polyvinyl butyrate nanoparticles with a shellac resin shell [61]. The shellac coat remains tightly arranged under gastric acidity, protecting the payload, and dissolves at the near-neutral pH of the lower gastrointestinal tract, releasing butyric acid in a sustained fashion at the colorectal site. Notably, this system was developed not for a gut disease per se but to act on the gut–bone axis in osteoporosis, illustrating both the versatility of colon-targeted release and the point that its therapeutic validation to date lies outside the CNS.

Prodrug and carrier-integrated designs blur the line between vehicle and drug. In an orally administered cellulose acetate butyrate (CAB) nanocarrier, the polymer is not merely a container but a colon-specific butyrate prodrug: its butyryl side chains are hydrolyzed by colonic alkaline pH and microbial esterases to liberate butyrate, while residual polymer fragments are fermented by the commensal microbiota into additional SCFAs [62]. Loaded with quercetin, the platform delivered synergistic anti-inflammatory effects in a murine colitis model. The design is elegant, but its endpoint—amelioration of colonic inflammation—again underscores that these systems are optimized for local gastrointestinal action.

Esterification to dietary fiber offers a human-validated route to site-specific colonic release. Inulin propionate ester (IPE), in which propionate is covalently bound to an inulin backbone, delivers SCFA to the proximal colon upon microbial fermentation of the carrier. In a randomized, controlled crossover study in humans, IPE increased colonic propionate delivery—confirmed by ^13^C tracer recovery—and reduced ad libitum energy intake relative to inulin control [52]. This trial is doubly relevant: it demonstrates that targeted colonic SCFA delivery is achievable in people, and it simultaneously exposes a barrier central to this review, since the free metabolite’s short half-life is precisely why covalent tethering to a fermentable carrier was necessary. A conceptually related prodrug, glyceryl tributyrate (tributyrin)—three butyrate molecules esterified to glycerol—is orally absorbed and hydrolyzed by lipases to release butyrate, reaching plasma concentrations unattainable with the free acid and advancing to Phase I clinical testing [51,63].

Nanoparticle carriers more broadly—polymeric, lipid, and biohybrid—supply tunable protection, controlled release, and the capacity to co-deliver a metabolite alongside a second agent, and the field has been reviewed comprehensively in the disease-agnostic setting [23].

Taken together, colon-targeted delivery is the mature wing of this field: multiple chemistries reliably place an intact microbial metabolite in the distal gut, and at least one has been validated in humans. The essential caveat for our purposes is that this maturity has been earned in the service of local gastrointestinal or gut–axis indications—colitis, osteoporosis, appetite regulation. For depression, colonic delivery is likely necessary but not obviously sufficient: where a therapeutic effect depends on a central action, an agent that stops at the gut wall has solved only the first half of the delivery problem. This motivates the second, far less settled front.

### 4.2. Brain-Targeted Delivery: An Emerging Frontier

Delivery to the brain must contend with the BBB, whose tight junctions and efflux systems exclude the overwhelming majority of small molecules and essentially all large or charged ones [64]. For microbiota-derived therapeutics, two routes are emerging. We present them in order of increasing engineering maturity: the biological principle that gut-derived vesicles can reach the brain, its adaptation into engineered carriers, and the still-scarce direct evidence in depression itself.

Principle: microbial extracellular vesicles can cross the BBB. Gut bacteria constitutively release nanoscale EVs—including outer membrane vesicles (OMVs) from Gram-negative species and membrane vesicles from Gram-positive species [65]—that carry proteins, lipids, and nucleic acids and participate in interkingdom signaling along the gut–brain axis. A systematic review of the primary literature concluded that bacterial EVs have been repeatedly documented crossing the BBB and subsequently modulating neurons, astrocytes, and microglia, although the mechanisms of passage remain incompletely defined and data specifically on human gut-microbiota EVs are still limited [66]; in one direct demonstration, outer membrane vesicles from a periodontal pathogen crossed the murine blood–brain barrier within hours of entering the circulation and drove inflammatory cytokine production [67]. This establishes the phenomenon on which any microbial–EV brain-delivery strategy depends: the gut can, in principle, dispatch vesicular cargo to the brain.

Engineering proof-of-concept: detoxified bacterial membranes as brain-targeting carriers. The most compelling demonstration that this natural tropism can be repurposed comes from work that turned a mechanism of bacterial neuroinvasion into a delivery tool. Escherichia coli K1 crosses the BBB during neonatal meningitis via an interaction between its outer membrane protein A (OmpA) and gp96 on brain microvascular endothelial cells. Chen and colleagues stripped the lipopolysaccharide (LPS) from the *E. coli* K1 outer membrane and used the detoxified membrane to coat nanoparticles, producing biomimetic carriers that crossed the BBB through OmpA–gp96-mediated transcytosis while exhibiting prolonged circulation and, critically, no concomitant toxicity [68]. Two features of this study bear directly on the present argument. First, the transport mechanism is transcellular transcytosis, which in principle does not require a disrupted barrier—a property whose significance for depression, where the barrier remains intact, we develop in Section 5. Second, the therapeutic efficacy was demonstrated in the setting of brain-metastatic cancer, exploiting gp96 overexpression on tumor cells; the delivery mechanism is thus BBB-intact-compatible, but the efficacy data were generated in a context with its own barrier peculiarities. This membrane-coating strategy complements a parallel line of work in which intact OMVs are themselves bioengineered as drug carriers [69]; surface display of brain-homing ligands—such as the LRP1-targeting peptide angiopep-2, an archetypal receptor-mediated-transcytosis vector [70]—together with attenuation to lower endotoxicity has since enabled engineered OMVs to ferry chemotherapeutic and nucleic-acid cargo across the BBB into glioblastoma [71]. The same surface-engineering logic is well established for mammalian extracellular vesicles [72,73], whose display of brain-homing ligands—such as the rabies-virus glycoprotein peptide—has delivered siRNA across the BBB to neurons [74]; mesenchymal-stem-cell-derived vesicles have likewise been engineered for targeted delivery to the ischemic brain [75], and strategies for targeting and crossing the BBB with EVs have been reviewed in detail [76]. We therefore read this work as a proof that detoxified microbial membranes can serve as brain-targeting carriers, while reserving the question of dose and effect in depression for Section 5.

Toward depression: bacterial–EV effects on mood-relevant biology, and a BBB-bypassing alternative. The strongest evidence that microbiota-derived vesicles can influence depression-relevant biology comes from probiotic bacterial EVs. EVs from *Lactobacillus plantarum* increased hippocampal brain-derived neurotrophic factor (BDNF) expression and produced antidepressant-like effects in mice [77], and EVs from Gram-positive and Gram-negative probiotics were subsequently shown to remediate stress-induced depressive behavior and to counteract chronic-stress-induced changes in neurotrophic factors [78]. These studies are the closest existing analogues to a microbial–EV antidepressant, and they anchor the therapeutic side of the paradox this review develops.

A distinct strategy sidesteps the BBB entirely. Intranasal administration exploits the olfactory and trigeminal pathways to transport cargo directly from the nasal cavity to the brain [79]. In a recent demonstration, extracellular vesicles derived from the microalga Chlorella vulgaris were formulated in a thermosensitive intranasal hydrogel and delivered to the hippocampus along olfactory routes; in LPS-induced and chronic-unpredictable-mild-stress models, the system produced rapid alleviation of depressive- and anxiety-like behavior, acting through astrocyte phenotype modulation, suppression of neurotoxic complement C3, and activation of the Nrf2–Pgc-1α antioxidant pathway [80]. Two points of precision warrant emphasis. These are microalgal extracellular vesicles, not gut-microbial or bacterial EVs, so the study exemplifies EV-based nose-to-brain delivery for depression rather than the bacterial–EV paradox specifically. And its route—intranasal—is BBB-bypassing rather than BBB-crossing, making it complementary to, not a substitute for, the systemic microbial–EV strategies above. Complementary to carrier design, physical methods can transiently and reversibly open the barrier itself: magnetic-resonance-guided focused ultrasound has safely and repeatedly opened the BBB in patients with Alzheimer’s disease, a clinically validated route that could be paired with microbiota-derived agents [81]. Edible extracellular vesicles such as bovine milk-derived exosomes are likewise attractive oral carriers, being inexpensive to source at scale, stable through gastrointestinal transit, and well tolerated across species [82]. Edible plant-derived exosome-like nanoparticles extend this further: ginger-derived nanoparticles survive gastrointestinal transit, are taken up by gut bacteria, and reshape microbiota composition and its indole-metabolite output [83]. Plant-derived exosome-like nanovesicles have been proposed more broadly as scalable, low-immunogenicity delivery nanoplatforms [84]. Milk-, plant-, and microalga-derived vesicles are nonetheless non-microbial comparator carriers: they illustrate what a bio-derived vesicle platform can achieve pharmaceutically, but they are neither microbiota-derived therapeutics nor evidence that bacterial EVs behave equivalently.

**Figure 3:**
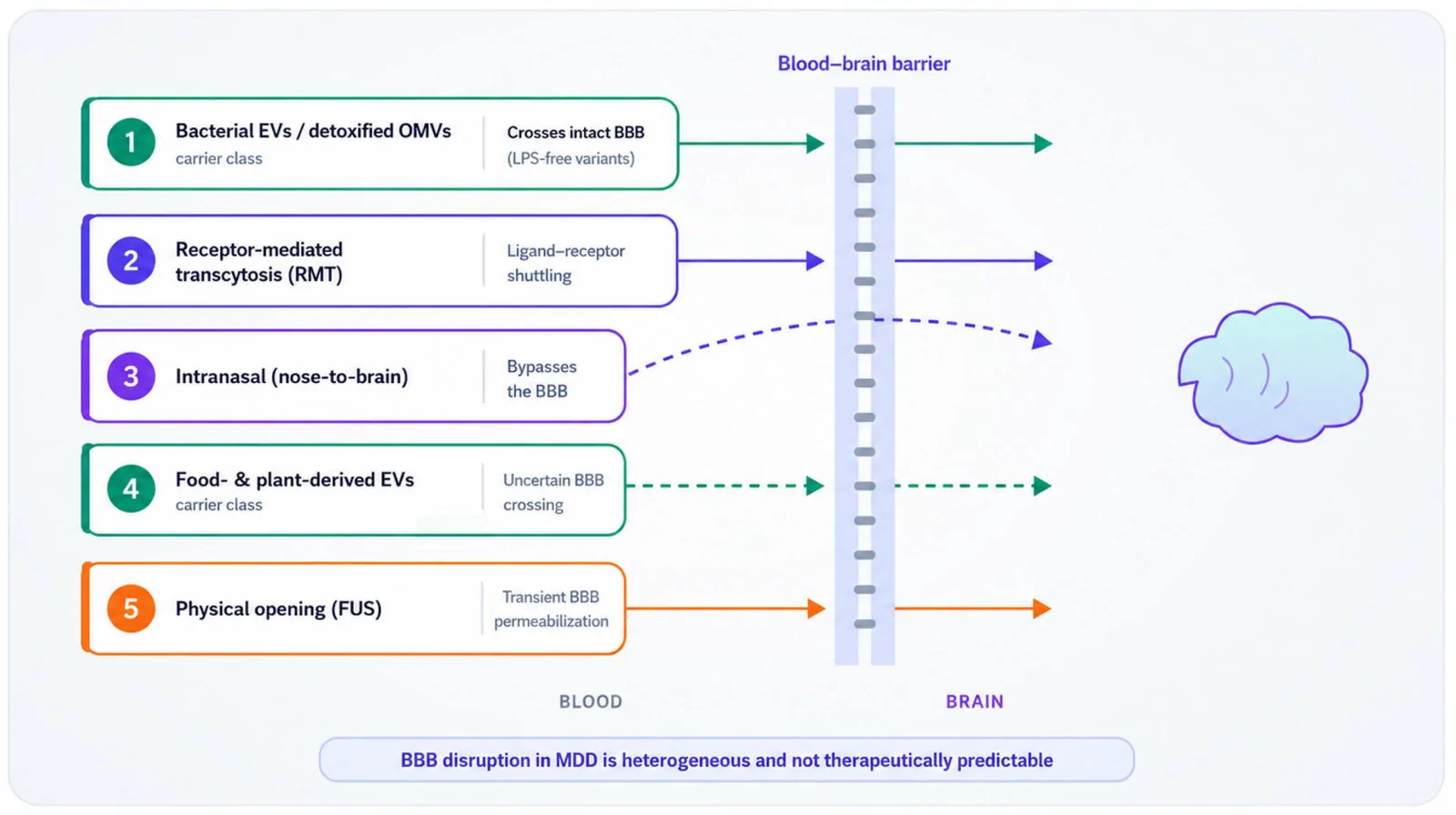
Brain-targeted delivery: an emerging frontier. Routes for moving microbiota-derived agents, or signals derived from them, across or around the blood–brain barrier (BBB): bacterial extracellular vesicles and LPS-free detoxified outer membrane vesicles that cross the intact barrier; receptor-mediated transcytosis via ligand–receptor shuttling; intranasal nose-to-brain transport that bypasses the BBB; food- and plant-derived EVs; and physical opening by magnetic-resonance-guided focused ultrasound (FUS). The enabling demonstrations lie largely outside depression; BBB disruption in depression is heterogeneous and not therapeutically predictable. Carrier types (vesicles) are distinguished from transport mechanisms (e.g., receptor-mediated transcytosis), and BBB-crossing routes from BBB-bypassing intranasal delivery. EV, extracellular vesicle; MDD, major depressive disorder; OMV, outer membrane vesicle. Non-microbial bio-derived extracellular vesicles are shown only as comparator delivery platforms and are not classified here as microbiota-derived therapeutics.

Read as a whole, brain-targeted delivery for microbiota-derived antidepressants is a frontier defined by a striking asymmetry: the biological capability is real and even engineerable, yet nearly every enabling demonstration was obtained in a non-depression indication—meningitis biology, brain-metastatic cancer, or plant–EV models—and the one route with direct antidepressant data bypasses the barrier the field most needs to cross. Converting these scattered results into a coherent, depression-validated delivery platform is the unresolved problem to which we turn next. The brain-targeting routes discussed here are summarized in Figure 3.

We therefore state the evidentiary position explicitly, and deliberately avoid any claim that microbial vesicles are superior brain-delivery vehicles. The engineering proof-of-concept studies above were performed in oncology, infection, or non-depression neurological settings, in which the barrier is frequently compromised and the therapeutic endpoint differs entirely from that of a chronic mood disorder; their success establishes feasibility of a transport mechanism, not efficacy for depression. Conversely, the studies with genuine antidepressant-like readouts either used probiotic bacterial EVs without demonstrating parenchymal cargo delivery, or bypassed the barrier by the intranasal route. At present, therefore, no study has shown quantitative brain exposure of a microbiota-derived vesicle together with target engagement and antidepressant efficacy in the same experiment, and comparative head-to-head data against established synthetic carriers are absent. Microbial vesicles are best described as a promising but unproven carrier class whose principal advantages—intrinsic barrier tropism and engineerable cargo—are matched by liabilities that synthetic carriers do not share. The evidence and limitations of these brain-delivery studies are summarized in Table 2.

**Table 2:** Reported brain delivery of microbiota-derived and bio-derived vesicles: evidence and limitations. “Brain delivery” should be judged by vascular perfusion, orthogonal labeling, quantitative brain exposure, and cargo target-engagement rather than whole-brain fluorescence alone. BBB, blood–brain barrier; BDNF, brain-derived neurotrophic factor; CUMS, chronic unpredictable mild stress; IP, intraperitoneal; IV, intravenous; NP, nanoparticle; OMV, outer membrane vesicle; PAMP, pathogen-associated molecular pattern.

Vehicle (Study)	Route	Model/Indication	BBB Status	Evidence for Brain Delivery	Depression Efficacy?	Key Limitation	Source Class	Evidence Context
Detoxified *E. coli* K1 membrane-coated NP [68]	IV	Brain-metastatic cancer	Often tumor-compromised	OmpA–gp96 transcytosis; prolonged circulation	No	Efficacy shown in tumor, not under intact BBB/depression	Microbial (engineered bacterial membrane)	Non-depression engineering proof-of-concept
Engineered OMV + angiopep-2 [71]	IV	Glioblastoma	Compromised	Cargo delivery to tumor	No	Tumor barrier; residual PAMP	Microbial (engineered bacterial OMV)	Non-depression engineering proof-of-concept
Periodontal-pathogen OMV [67]	Systemic	Healthy mice	Intact	Brain entry within hours; pro-inflammatory	No (pathological)	Pathogen OMV; harm, not benefit	Microbial (bacterial OMV)	Adjacent CNS (pathological)
Probiotic bacterial EVs [77,78]	IP/oral	Chronic-stress depression models	Presumed intact	Antidepressant-like behavior; ↑ hippocampal BDNF	Yes (rodent)	Parenchymal EV-cargo delivery not directly shown	Microbial (bacterial EV)	Depression-specific
Chlorella vulgaris (microalgal) EV, intranasal [80]	Intranasal (bypass)	LPS/CUMS depression models	Bypassed	Hippocampal delivery; behavioral rescue	Yes (rodent)	Microalgal (non-bacterial) EV; rodent nasal anatomy	Non-microbial comparator	Depression-specific
Milk/ginger edible EVs [82,83]	Oral	Gastrointestinal/microbiota	n/a (peripheral)	Gut uptake; microbiota modulation	No	Peripheral action; not brain delivery	Non-microbial comparator	Non-depression comparator

### 4.3. Pharmaceutical and Biopharmaceutical Criteria for Translational Delivery

Transferability of a delivery platform cannot be inferred solely from tissue localization or therapeutic efficacy. For orally delivered microbiota-derived metabolites, the relevant pharmaceutical endpoints include stability during gastrointestinal transit, site-specific release, local luminal exposure, systemic bioavailability where a systemic action is intended, C_max_, T_max_, AUC, elimination half-life, and exposure–response relationships. For centrally directed systems, qualitative whole-brain fluorescence alone provides insufficient evidence of delivery; quantitative plasma and brain exposure, vascular perfusion or capillary depletion, label stability, cargo-specific detection, and pharmacodynamic target engagement should be reported whenever feasible. A recurring weakness of the literature synthesized here is that these endpoints are seldom reported together, which allows engineering feasibility to be conflated with pharmacological plausibility.

Formulation development should additionally define critical quality attributes appropriate to each platform, including particle-size distribution, polydispersity, surface charge, drug loading or encapsulation efficiency, release kinetics in biorelevant media, physicochemical stability, and batch-to-batch reproducibility. For bacterial extracellular vesicles and outer-membrane-vesicle-derived systems, source strain, culture conditions, isolation and purification procedures, particle concentration, cargo composition, residual endotoxin and other pathogen-associated molecular patterns, sterility, and storage stability represent additional product-specific variables that have no counterpart in conventional small-molecule formulation.

These requirements are particularly important for depression because treatment is likely to involve repeated or chronic administration. Repeated-dose studies should therefore characterize systemic clearance, organ biodistribution, brain exposure, cytokine responses, complement activation where relevant, anti-vesicle immune responses, organ toxicity, and changes in exposure after repeated dosing, rather than relying on acute single-dose tolerability. Table 3 summarizes these criteria across the platform classes considered in this review.

Recent formulation advances are especially relevant to the nose-to-brain route, which is the only route in this review with direct antidepressant-like evidence. Contemporary intranasal systems combine nanocarriers with vehicles designed to prolong nasal residence: thermoresponsive and in situ gelling hydrogels, mucoadhesive polymers, and lipid- or polymer-based nanoparticles dispersed within them, with performance governed by viscosity and gelation behavior, sprayability and droplet size, deposition in the olfactory region, and the competition between mucoadhesion and mucociliary clearance [85,86]. Biopharmaceutical factors—particle size and surface charge, mucus interaction, and enzymatic stability in nasal tissue—determine how much of an administered dose engages the olfactory and trigeminal pathways rather than being cleared or absorbed systemically [85]. The microalgal-vesicle study discussed above is consistent with this design logic, since it used a thermosensitive intranasal hydrogel rather than a vesicle suspension alone; the corollary is that rodent nasal anatomy and the proportional area of olfactory epithelium differ substantially from the human situation, so nose-to-brain efficiency should not be extrapolated directly [86].

## 5. Current Challenges: The Paradox of the Pathological Carrier

**Figure 4:**
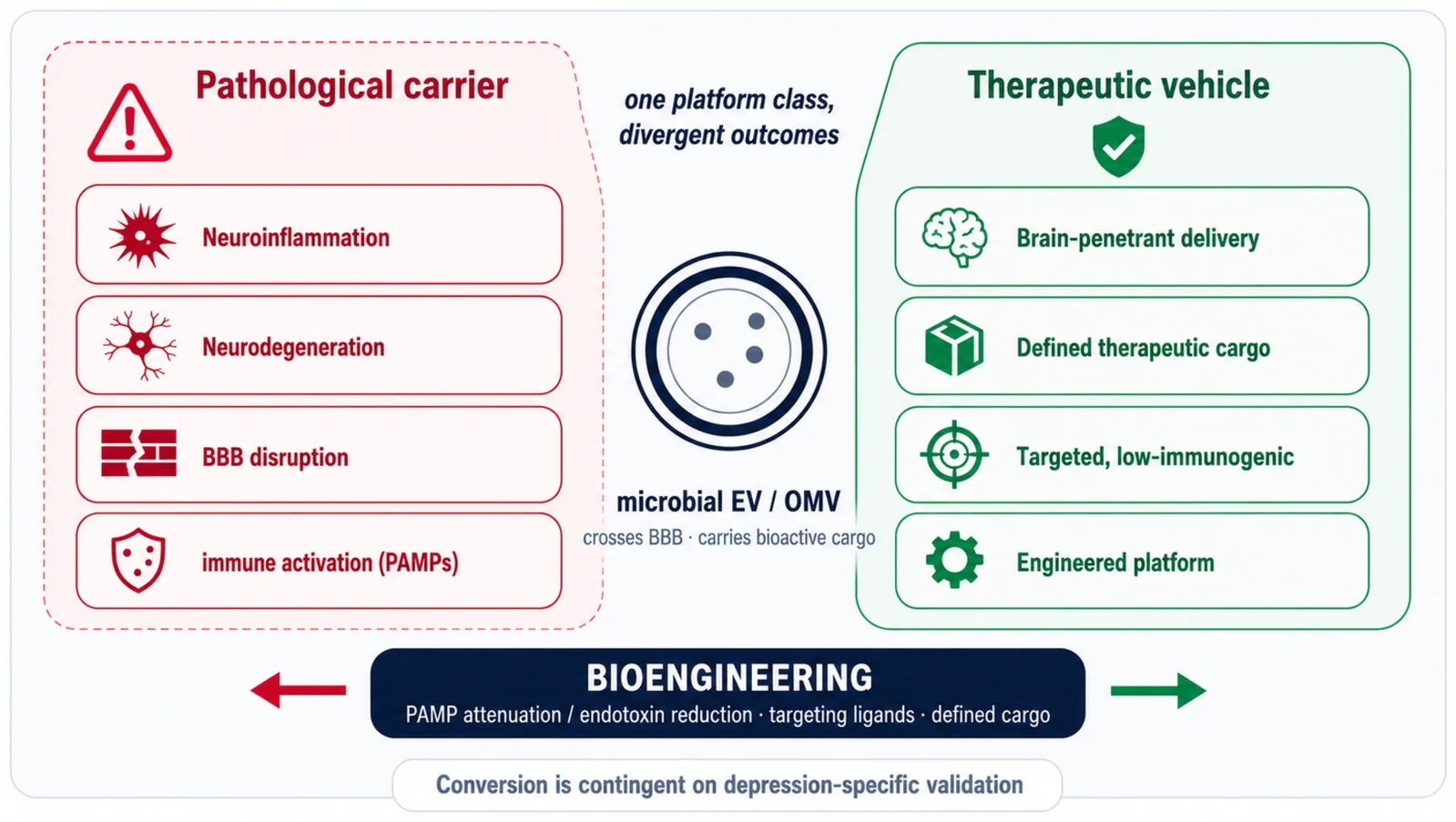
The microbial extracellular vesicle risk–opportunity continuum. Selected microbial vesicles have shown brain exposure in preclinical models and carry bioactive cargo. The paradox is not that one identical vesicle has two interchangeable fates, but that the outcome depends on source, cargo, dose, route, and host context: in the dominant literature these vesicles appear as drivers of neuroinflammation and neurodegeneration (**left**), whereas de-risking and engineering—PAMP attenuation/endotoxin reduction, targeting-ligand display, and defined cargo—could position them as therapeutic vehicles (**right**), contingent on depression-specific validation. BBB, blood–brain bar-rier; EV, extracellular vesicle; OMV, outer membrane vesicle; PAMP, pathogen-associated molecular pattern.

The delivery strategies of Section 4 make the field’s promise concrete, but they also expose its defining tension. One of the more experimentally documented biological routes to brain exposure considered here—microbial extracellular vesicles—is also the route most deeply associated with harm. This is the paradox that, in our view, defines the current landscape and organizes its unresolved problems (Figure 4).

The carrier is also the culprit. Gut microbial EVs have long been described as a double-edged sword for the gut–brain axis [87,88]. The same properties that make them attractive vehicles—their capacity to cross biological barriers, including the BBB, and to deliver bioactive protein and nucleic-acid cargo to neurons, astrocytes, and microglia—are the properties through which they have been implicated as causative agents of neuropathology. Bacterial EVs have been documented crossing the BBB and eliciting proinflammatory signaling in the brain [66], and gut microbial EVs have been implicated more broadly in the neuroinflammation and neurodegeneration surrounding stroke, Parkinson’s disease, and Alzheimer’s disease. This wider literature warrants care: some of its most-cited evidence concerns microbiome- or pathogen-associated pathology rather than EV-mediated transport per se—gut bacteria and their metabolites are required for α-synuclein pathology and microglial activation in Parkinson models [89], and Porphyromonas gingivalis and its gingipains are linked to Alzheimer pathology [90], yet neither establishes bacterial EVs as the causal vehicle. The more directly EV-relevant claim is that bacterial membrane vesicles may link peripheral bacterial infection to central amyloid and tau pathology [87,91]. In other words, the literature that would supply a microbial–EV delivery platform for depression is, at present, largely a literature about how microbial EVs cause brain disease. Repurposing this route is therefore not a matter of straightforward adoption; it requires engineering out the very activity for which these vesicles are best known—as the LPS-free, detoxified outer-membrane approach of Chen et al. [68] begins to demonstrate, but which remains far from a validated therapeutic standard. Indeed, not all microbial vesicles are harmful: outer membrane vesicles from the probiotic Escherichia coli Nissle 1917 exert anti-inflammatory effects and reinforce the intestinal barrier in experimental colitis [92], underscoring that the prevailing pathological framing reflects which vesicles have been studied rather than an intrinsic property of the platform.

The efficacy evidence lies in the wrong disease. A related and easily overlooked problem is that the most persuasive brain-delivery demonstrations were obtained in conditions unlike depression. The detoxified *E. coli* K1 carrier established its therapeutic payoff in brain-metastatic cancer, exploiting tumor-associated gp96 overexpression [68]; much of the broader engineered-vesicle and OMV brain-delivery literature likewise targets glioma and glioblastoma [69,71]. These are settings in which the BBB is frequently compromised—partially disrupted by the tumor and its microenvironment—which materially eases delivery. Depression is not such a setting: the barrier is neither reliably disrupted nor dependably exploitable, and cannot be assumed permeable. Honesty about the state of the field requires acknowledging that importing brain-tumor-derived delivery data into depression risks overestimating what these carriers will achieve when the barrier they must cross is whole. The encouraging counterpoint is mechanistic—the OmpA–gp96 transcytosis route is, in principle, a transcellular mechanism that does not depend on a leaky barrier—but “in principle” is not “in a depression model,” and closing that gap is a specific, testable priority rather than an assumption to be waved through.

Dose–response, repeated-dose immunogenicity, and biodistribution remain undefined. Beyond the paradox itself, the practical parameters of a microbial–EV or microbial–metabolite antidepressant remain almost entirely unspecified. There is no established dose–response relationship for centrally acting microbiota-derived agents delivered by these routes, and depression—typically requiring sustained rather than acute treatment—raises questions of chronic administration that acute proof-of-concept studies cannot answer. The safety concerns are not hypothetical: residual immunostimulatory components such as LPS, the immunogenicity of repeatedly administered bacterial-derived material, and off-target biodistribution—systemically administered EVs accumulate predominantly in the liver, spleen, and lungs, with only a small targeted fraction reaching the brain [93,94]—all bear directly on a chronic indication. Reviews of bacterial metabolites as immunomodulators have cataloged precisely these formulation, stability, safety, and off-target challenges as the gating issues for translation [95].

Individual microbiome variability threatens reproducibility. A challenge peculiar to this field is that both the substrate and, in some strategies, the vehicle are functions of an individual’s microbiome, which varies substantially between people and over time. Strategies that rely on the resident microbiota to ferment a carrier or to generate the active metabolite inherit that variability directly, complicating dose standardization and reproducibility in ways that conventional small-molecule pharmacology does not face. This argues for delivery designs that are robust to inter-individual microbial differences, or alternatively for approaches explicitly personalized to an individual’s microbiome profile.

Depression-specific clinical data are essentially absent. Finally, and underlying all of the above, the direct evidence base in depression is thin. The strongest depression-relevant results come from probiotic–EV studies in rodent models [78] and from a plant–EV intranasal system in mouse depression models [80]; there is, as yet, no clinical validation of a microbiota-derived therapeutic delivered by these routes for MDD. The field’s central claims about delivery are therefore, for now, extrapolations from adjacent diseases and preclinical models—well-motivated extrapolations, but ones whose translation to human depression remains to be demonstrated.

Framed together, these challenges are not a miscellany but facets of a single situation: a delivery route of real capability, developed almost entirely in the study of disease, awaiting the deliberate engineering and depression-specific validation that would convert it from a pathological mechanism into a therapeutic platform. That conversion is the subject of Section 6.

### Long-Term Immunogenicity, Biodistribution, and Manufacturing Reproducibility

Three requirements will determine whether any microbiota-derived vesicle can be given repeatedly to patients, and each is currently under-reported. The first is long-term immunogenicity. Bacterial vesicles are, by construction, assemblies of pathogen-associated molecular patterns: lipid A and lipopolysaccharide in Gram-negative outer membrane vesicles, and lipoteichoic acid, peptidoglycan fragments, and lipoproteins in Gram-positive vesicles, together with residual nucleic acids. Attenuating lipid A lowers but does not abolish innate stimulation, and single-dose tolerability in rodents is a weak predictor of what repeated dosing will produce. Chronic administration raises the prospect of anti-carrier antibody formation with accelerated clearance on re-dosing, complement activation and infusion reactions, cytokine release, and—most relevant here—low-grade systemic inflammation, which is precisely the mechanism implicated in inflammatory phenotypes of depression. A therapy that delivers an antidepressant payload while sustaining an inflammatory tone would be self-defeating. Repeated-dose studies should therefore report endotoxin content by a validated assay, cytokine time-courses, anti-carrier immunoglobulin titers, complement split products, organ histopathology, and markers of neuroinflammation.

The second is biodistribution. Systemically administered vesicles are cleared predominantly by the liver and spleen, and the fraction reaching the brain parenchyma is typically a small percentage of the injected dose. Reporting should accordingly be quantitative and route-specific: percentage of injected dose per gram of tissue across organs, plasma concentration–time profiles, and brain exposure determined after vascular perfusion or capillary depletion, with orthogonal labeling to exclude dye artifacts. Distribution should be examined under the relevant disease state rather than only in healthy animals, since chronic stress and inflammation alter both barrier properties and phagocyte activity, and in both sexes.

The third is manufacturing reproducibility. Vesicles are not molecularly defined entities, and their cargo varies with source strain, passage, growth medium, oxygen tension, growth phase, and isolation method; a change in any of these can alter potency and immunogenicity without any change to the nominal product. Chemistry, manufacturing, and controls for such a product must therefore specify a banked and genomically characterized production strain screened for virulence and antimicrobial-resistance determinants, defined and preferably animal-component-free culture conditions, a scalable purification train with documented removal of free lipopolysaccharide, nucleic acids, and media components, and release specifications covering particle concentration and size distribution, protein-to-particle ratio, identity markers, endotoxin, sterility, residual host nucleic acid, potency in a functional assay, and stability under defined storage. Reporting standards for extracellular vesicles provide a starting framework, but were developed principally with eukaryotic vesicles in mind; bacterial vesicle preparations require the additional strain-, contaminant-, and PAMP-related attributes listed above [96]. Until these three areas are addressed with the same rigor now applied to the delivery chemistry, claims of clinical readiness for depression are premature.

## 6. Future Directions

The through-line of this review is that a route developed almost entirely in the study of disease must be deliberately re-engineered into a therapeutic platform. That conversion defines the field’s agenda, and several directions follow from it.

**Figure 5:**
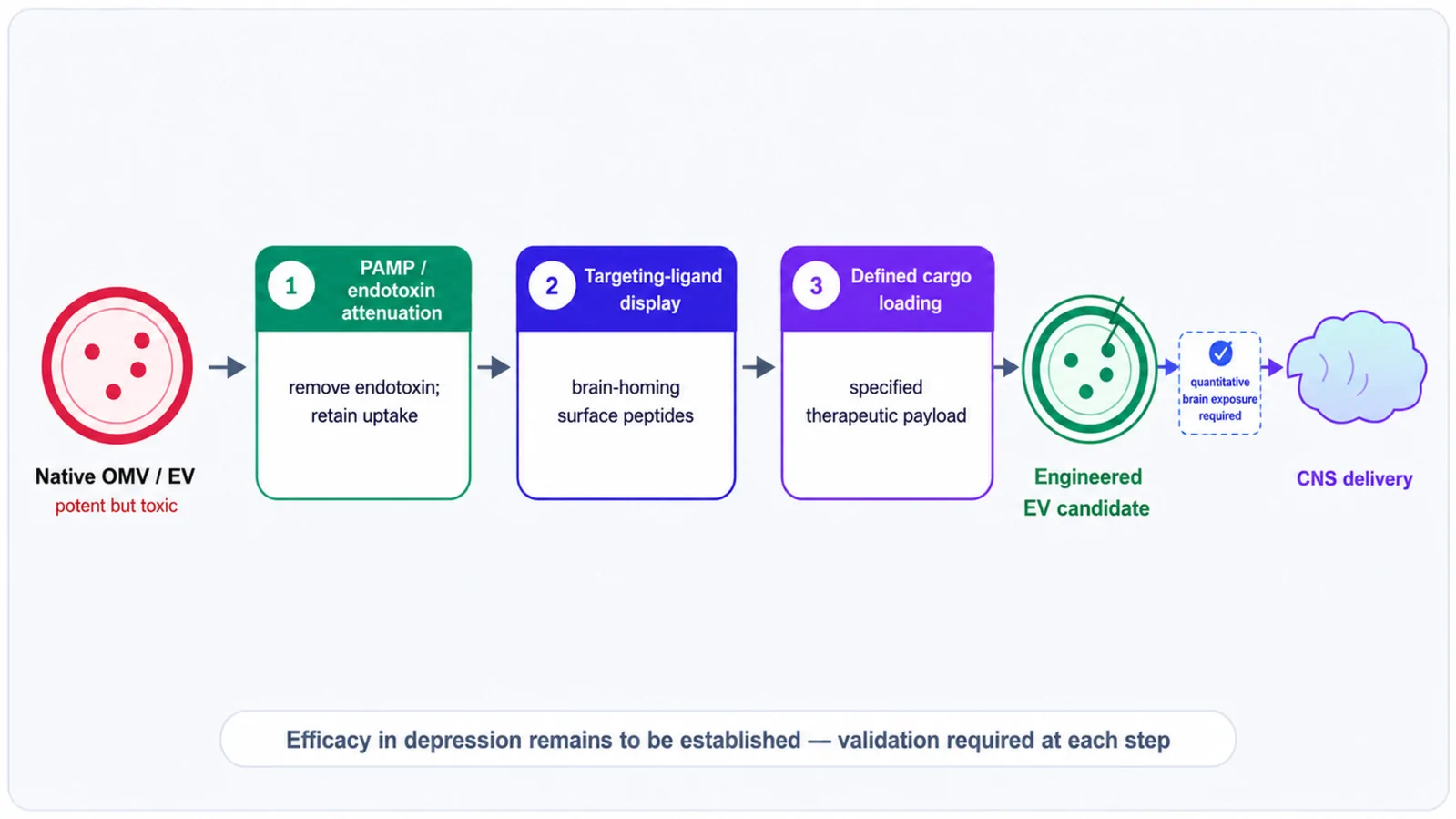
Bioengineering the microbial vesicle. A staged pipeline to convert a native, potent-but-toxic outer membrane vesicle (OMV) or extracellular vesicle (EV) into an engineered EV candidate: (1) PAMP/endotoxin attenuation to reduce immunogenicity while retaining barrier-crossing uptake; (2) display of brain-homing targeting ligands; and (3) loading of defined therapeutic cargo. Quantitative brain exposure must be demonstrated before central delivery is claimed, and efficacy in depression remains to be established; validation is required at each step. CNS, central nervous system; PAMP, pathogen-associated molecular pattern.

Bioengineering the vehicle. The first requirement is to subtract harm and add control. Detoxification is the proof that this is feasible: removing lipopolysaccharide from a bacterial outer membrane retained the membrane’s barrier-crossing capacity while eliminating its toxicity [68]. Because immunostimulation is not confined to the lipopolysaccharide of Gram-negative vesicles—Gram-positive vesicles carry lipoteichoic acid, peptidoglycan-associated components, and lipoproteins—the more accurate objective is broad pathogen-associated-molecular-pattern (PAMP) attenuation and endotoxin reduction rather than LPS removal alone. Beyond detoxification, the toolkit now under development includes engineering vesicle surfaces with targeting ligands to bias biodistribution toward the brain, loading defined therapeutic cargo in place of native pathological content, and attenuating residual immunogenicity. Synthetic-biology and metabolic-engineering approaches point further still, toward designer vesicles produced to specification rather than harvested and repurposed [95,97]. Indeed, engineered gut commensals already function as in vivo metabolic factories: an engineered Escherichia coli Nissle strain that degrades phenylalanine in the gut has advanced from rodent and primate models toward clinical testing, illustrating that microbes can be programmed to produce or consume defined metabolites at the delivery site [98]. These engineered strains fall within the emerging class of live biotherapeutic products, whose distinct regulatory and manufacturing requirements are now being defined [99]. The conceptual shift here is from using microbial EVs as found to manufacturing them as intended. The staged bioengineering pipeline is outlined in Figure 5.

Precision delivery informed by the individual microbiome. Because both the payload and, in some designs, the vehicle depend on a person’s resident microbiota, inter-individual microbial variability is not merely a confounder to be minimized but a variable that could be exploited. Delivery strategies matched to an individual’s microbiome profile—selecting carriers or prodrug chemistries whose activation suits that person’s fermentative and enzymatic capacity—offer a route to the dose standardization that population-level designs struggle to achieve [95], extending the psychobiotic concept toward precision, individually tailored interventions [100,101]. Computational and machine-learning approaches to carrier design and to predicting metabolite–microbiome interactions are a natural, if still early, complement to this personalization [102].

Standards for clinical translation. None of the above reaches patients without a characterization and safety infrastructure that the field does not yet possess. Standardized isolation and characterization of microbial EVs remain unresolved, and the inconsistency this introduces has been repeatedly flagged as a barrier to reproducible research [66,96]. Translation for a chronic indication such as depression will additionally demand established dose–response relationships, long-term safety data addressing immunogenicity and off-target biodistribution, and formulation stability suitable for sustained use [95]. These are unglamorous requirements, but they, rather than any single delivery breakthrough, are likely to determine whether microbiota-derived antidepressants become clinical realities.

Most importantly, every strategy above must ultimately be validated in depression itself rather than inferred from adjacent indications. The defining task is not only to build better carriers but to demonstrate, under the intact-barrier conditions of depression, that they deliver a therapeutic effect.

## 7. Conclusions

We have argued for a change of emphasis: from asking which microbiota-derived molecules might treat depression to asking how they can be delivered to where they must act. Under this lens the field’s structure comes into focus. Colon-targeted delivery is a maturing engineering discipline, with chemistries that reliably place intact microbial metabolites in the distal gut and at least one platform validated in humans—but validated for local gastrointestinal and systemic indications, not yet for a central effect in depression. Brain-targeted delivery is a genuine but early frontier, in which microbial extracellular vesicles can demonstrably reach the brain and even be engineered to do so, yet whose enabling demonstrations were obtained in meningitis biology, brain-metastatic cancer, and plant–EV models rather than in depression.

Binding these observations together is the paradox we take to be the field’s defining feature: microbial EVs have demonstrated the capacity to reach the brain in selected preclinical settings, but the same vesicle class is also strongly associated with neuroinflammatory signaling. The central opportunity—and the central unresolved problem—is to convert this pathological route into a safe, engineered, depression-validated delivery platform. Delivery, in short, is a major—though not the only—bottleneck standing between microbiota-derived therapeutics and the clinic, and at the same time one of the most compelling opportunities the field affords. Progress will require validating site of action, quantitative exposure, target engagement, chronic efficacy, and manufacturing reproducibility in parallel; the framework proposed here is intended to guide route-specific testing rather than to imply that any current platform is ready for clinical development in depression. Microbial EVs provide biologically plausible and experimentally demonstrated routes for brain exposure in selected preclinical settings, but their therapeutic use in depression remains unvalidated. Their value therefore lies not in established superiority over other delivery platforms, but in the possibility that a naturally occurring interkingdom transport mechanism could be de-risked and engineered for controlled therapeutic delivery.

## Figures and Tables

**Table 3:** Pharmaceutical development criteria for microbiota-associated delivery platforms. CQA, critical quality attribute; PK, pharmacokinetic; RES, reticuloendothelial system; PAMP, pathogen-associated molecular pattern.

Platform	Critical Quality Attributes	Key Biopharmaceutical/PK Endpoints	Major Translational Limitation
Colon-targeted metabolite/prodrug	GI stability, activation specificity, release kinetics, drug content	Colonic exposure, T_max_, systemic AUC if required	Variable microbiota-dependent activation
Oral polymeric/lipid nanoparticle	Size, PDI, zeta potential, loading, release, mucus interaction	GI retention, absorption, oral bioavailability	Mucus clearance, premature release
Microbial EV/OMV	Source strain, particle number and size, cargo, PAMP/endotoxin burden, purity	Clearance, t_1_/_2_, organ biodistribution, quantitative brain exposure	Immunogenicity, RES uptake, manufacturing variability
Detoxified membrane-coated nanoparticle	Membrane composition, ligand density, endotoxin, size, stability	Circulation time, brain exposure, target engagement	Loss of biological function during detoxification and scale-up
Intranasal EV/nanocarrier/hydrogel	Viscosity, gelation, mucoadhesion, particle size, sprayability, release	Nasal residence, brain exposure, systemic spillover	Mucociliary clearance, rodent–human anatomical differences

## Data Availability

No new data were created or analyzed in this study. Data sharing is not applicable to this article.
